# Inorganic and Organic Selenium Speciation of Seleno-Yeasts Used as Feed Additives: New Insights from Elemental Selenium Determination

**DOI:** 10.1007/s12011-023-03633-z

**Published:** 2023-03-18

**Authors:** Mohammed A. Hachemi, Denise Cardoso, Michele De Marco, Pierre-André Geraert, Mickael Briens

**Affiliations:** Adisseo France S.A.S., 10, Place du Général de Gaulle, 92160 Antony, France

**Keywords:** Selenium yeasts, Elemental selenium, Inorganic selenium, Selenomethionine, Speciation

## Abstract

Seleno-Yeasts (SY) used as feed additives are known to contain different Selenium (Se) species. Seleno-Yeasts has been shown, on previous analytical methods, to contain selenomethionine (SeMet), selenocysteine (SeCys), selenate (Se^IV^) and selenite (Se^VI^), and various other organic and inorganic Se forms identified but rarely quantified. A new advanced method has allowed elemental Se (Se^0^), an inorganic Se species, to be quantified, thereby obtaining better insight into the proportion of inorganic Se in SY products. The study aimed to quantify the Se^0^ in SY products and assess the proportion of inorganic Se in SY. The Se speciation of 13 fresh commercials SY from different suppliers and batches, was assayed for the total Se, inorganic Se species (Se^IV^, Se^VI^ and Se^0^), and organic Se species (SeMet and SeCys). Results on total Se were in line with the expected Se concentrations for all evaluated samples. The proportion of Se present as Se^0^ ranged from 3.6% to 51.8%. The quantity of Se^0^ in the SY products, added to Se^IV^ and Se^VI^, indicated an average proportion of inorganic Se of 14.2% for the 13 analyzed SY products. The proportion of Se as SeMet ranged from 19.0% to 71.8%, (average of 55.8%), and a large variability in the SeMet content was observed. The SeCys content was also variable, with an average of 3.8%, relative to the total Se. In conclusion, advances in the analytical characterization have revealed that SY products can have a significantly high proportion of inorganic Se, which could affect the bioavailability of Se from SY supplements and explain their variable and lower bio-efficacy than pure SeMet supplements, such as hydroxy-selenomethionine.

## Introduction

Selenium (Se) is an essential trace element for all animal species. A deficiency in Se is associated with poor health, increased susceptibility to various diseases and decreased productive and reproductive performances of animals [1]. To avoid the consequences of a Se deficiency and to meet the Se requirements, its supplementation in feeds has become a common practice worldwide. Se supplements already exist commercially in inorganic forms, such as sodium selenite (Se^IV^) and selenate (Se^VI^), and in organic forms, such as seleno-yeasts (SY) or pure forms of selenomethionine (SeMet or OH-SeMet). In general, the inorganic forms of Se are absorbed less effectively as they have a far lower bio-efficacy than organic forms [2, 3]. When comparing organic Se sources, their bio-efficacy has been attributed to their SeMet proportion, since it is the only Se species that can be stored in tissues and build up a Se reserve in the body [2, 3]. Seleno-yeasts often shows a lower and variable bio-efficacy than pure SeMet [3], due to the variable content of SeMet in SY products. However, there is still limited knowledge about the other Se species present in SY products, and their characterization still requires further investigation. Thus, Se speciation in SY is necessary to obtain a better understanding of its metabolism and biological significance in animal nutrition.

Seleno-yeasts are produced by the aerobic fermentation of yeasts, mainly the *S. cerevisiae* yeast strain, in a medium that contains sugar beet pulp or cane molasses, vitamins, nutritional salts and sodium selenite as the Se source [4, 5]. Briefly, the enzymatic systems in growing yeast are not able to distinguish between Se and Sulfur (S), due to their similar chemical and physical properties [6]. Therefore, during the fermentation process, Se is used for SeMet synthesis in a similar way to S during the synthesis of methionine. Commercial SY products contain between 1,000 to 3,000 mg of Se/kg, which is composed of SeMet and other Se species that have formed as secondary metabolites during the fermentation process [2, 7]. Seleno-yeasts are known to have a complex composition of Se species [7–9]. In fact, over 180 Se compounds have recently been identified in 5 Seleno-yeasts products, and the authors suggested that different SY could vary in efficacy due to differences in the compound composition of Se [10]. According to the European commission, SY should contain at least 97% of organic Se relative to the total Se, and a minimum of 63–70% of Se as SeMet [11, 12]. The proportion of inorganic Se has been indicated to be less than 2% [13–16]. Likewise, FDA [17] and AAFCO [18] have regulated SY related to animal feed additives; inorganic Se should be < 2% of the total Se. However, commercial SY products are facing several issues, including inconsistencies in SeMet concentrations [8, 19] and unreachable mass balances between quantified Se species and total Se concentration in SY [8, 19]. To date, the proportion of inorganic Se has been characterized by Se^IV^ and Se^VI^, which often represent less than 2% of the total Se content of the SY products, and the remaining Se species are considered to be organic, including any unaccounted form of Se compounds. Nevertheless, recent advances in analytical techniques have allowed these Se forms to be further characterized. Vacchina et al. [20] developed a new method to quantify elemental Se (Se^0^) in SY products. Elemental Se is an inorganic form of Se that had previously been unquantified in SY, even though it was suspected of being generated through a fermentation process in ruminants fed selenite [21]. Therefore, the objective of this study has been to quantify Se^0^ in different commercial SY products used in animal nutrition by using an advanced analytical approach and to assess the contained proportion of organic/inorganic Se on the basis of these analytical advances.

## Material and Methods

### Seleno-Yeast Samples

Thirteen fresh SY samples (i.e. within expiration date) from different commercial products and batches were obtained from the market. The considered SY samples are from very common strains of Se enriched yeast, including CNCM I-3060, CNCM I-3399, NCYC R397, NCYC R645 and NCYC R646, which are authorized for animal feeds throughout the world. The labeled total Se content of these products ranged between 1,000 and 3,000 mg/kg.

### Procedure Analysis

All the samples were sent to the UT2A lab (Ultra-Trace Analyses Aquitaine, Pau, 64,053, France) to establish the total Se content and conduct the speciation analysis. Se-enriched yeast certified reference material (SELM-1, NRCC, Canada) was used for a quality control to determine the total Se and SeMet. All the analysis were performed in duplicate.

### Standards

For total Se determination, a multi-elemental stock standard containing 100 μg/mL of Se (MISA-4, CPAchem, Trappes, France) was used. For speciation analysis, 1000 μg Se/mL Se^IV^ and Se^VI^ were purchased from CPAchem (Trappes, France) and stock solutions of SeMet (1000 µg Se/mL) were prepared by dissolving the proper amount of the salt in ultrapure water. Elemental Se standard (1000 µg/mL) was prepared by dissolving Se^0^ in 1 M sodium sulfite (for details, see Vacchina et al. [20]).

### Total Se Determination

The determination of the total Se in the SY samples was performed using the method described by Bierla et al. [22]. Briefly, the samples were mineralized with a mixture of HNO3/H2O2 on a hot plate and then analyzed by means of Inductively Coupled Plasma Atomic Emission Spectrometry (ICP-AES). Quantification of 10 mg/kg could be reached with an intermediate fidelity of 2%.

### Determination of Selenomethionine, Selenite and Selenate

The quantification of SeMet, selenite (Se^IV^) and selenate (Se^VI^) in the SY samples was performed using high-performance liquid chromatography, coupled with inductively coupled plasma mass spectrometry (HPLC-ICP-MS) after double proteolytic digestion of the yeast, as described by Goenaga-Infante et al. [23] and Bierla et al. [22].

### Selenocysteine Determination

Selenocysteine is an unstable molecule that is highly susceptible to oxidation [24]. Therefore, before digestion of the proteins by proteolysis, the selenocysteine was stabilized by means of carbamidomethylation, as described by Dernovics et al. [25] and Bierla et al. [22]. The proteolytic digestion and quantification of CAM-SeCys was then performed as for SeMet.

### Elemental Se (Se^0^) Determination

The analysis of Se^0^ was conducted using the new advanced method developed by Vacchina et al. [20]. In short, the method consists of extracting Se^0^ from SY samples using an excess of sodium sulfite to transform insoluble Se^0^ into soluble selenosulfate. Selenosulfate, which is representative of the Se^0^ content in SY, was then quantified by means of HPLC-ICP-MS. The analytical figures of merit of the methods have shown recovery levels ranging from 93 to 101% and within and between-run precision below 8%. The quantification limit of Se^0^, when using this method, is 1 mg/kg.

## Results and Discussion

Seleno-Yeast was the first approved source of organic Se to be used as a feed additive for farm animals. Although the speciation of SY has already been performed, its full composition still needs a more detailed elucidation. In this study, the Se speciation of different commercial SY products has been explored using state-of-the-art and new analytical methods.

## Quantification of the Total Se in Seleno-Yeasts

The total Se content in the SY samples was found to be in accordance with the labeled values, that is, between 1,000 and 3,000 mg/kg (Table 1). High levels of Se induce toxicity in yeasts, which limits their capacity to ferment and convert selenite into different Se species. This explains why most commercial SY products have a maximum of 3,000 mg of Se/kg [2, 8, 24].

**Table 1:** The total Se and elemental Se (Se0) contents of various fresh commercial Seleno-yeast (SY) products

Commercial Seleno- yeast samples	Total Se content (mg/kg) ± uncertainty*	(Se^0^) content (mg/kg) ± uncertainty*	(Se^0^) content as % of total Se
SY-A	2379 ± 24	206 ± 2	8.7%
SY-B	2106 ± 21	116 ± 1	5.5%
SY-C	2024 ± 115	156 ± 2	7.7%
SY-D	2018 ± 20	73 ± 9	3.6%
SY-E	1897 ± 51	247 ± 6	13.0%
SY-F	1281 ± 41	664 ± 46	51.8%
SY-G	2143 ± 185	641 ± 67	29.9%
SY-H	2282 ± 101	196 ± 2	8.6%
SY-I	2794 ± 28	255 ± 6	9.1%
SY-J	3036 ± 113	345 ± 6	11.4%
SY-K	2101 ± 21	177 ± 30	8.4%
SY-L	2080 ± 20	377 ± 4	18.1%
SY-M	2079 ± 5	139 ± 3	6.7%

^*^uncertainty was calculated on the basis of two replicates

## Quantification of Selenite (Se^IV^) and Selenate (Se^VI^)

**Fig. 1:**
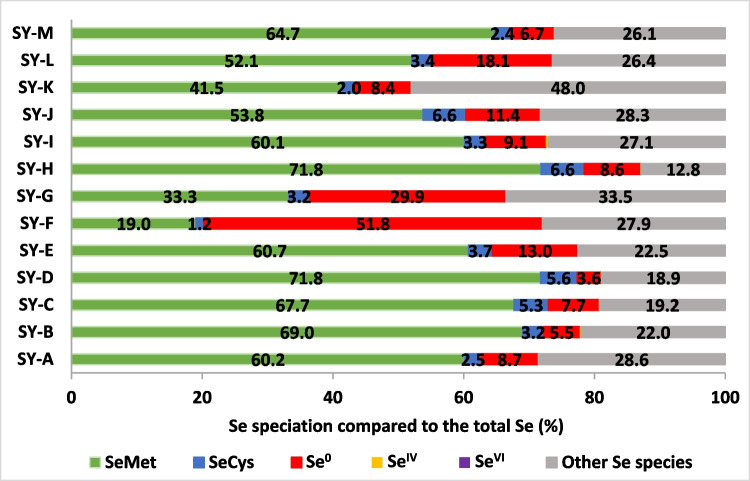
Se speciation compared with the total Se (%) of various fresh commercial Seleno-yeast (SY) products. The concentrations of Selenomethionine (SeMet), Selenocysteine (SeCys), Elemental Se (Se^0^), Selenite (Se^IV^), Selenate (Se^VI^) and other Se species are expressed as the % of the total Se. The analysis was conducted using the HPLC-ICP MS technique for all the Se species and ICP AES for the total Se. Limit of quantification (LOQ) are 10, 0.2, 1, 1, 0.2 and 0.5 mg/kg for total Se, SeMet, SeCys, Se^0^, Se^IV^ and Se^VI^, respectively. The proportions of Se^IV^ were: 0.1% for SY-A, 0.2% for SY-B, 0.1% for SY-C, 0.1% for SY-D, < LOQ for SY-E, < LOQ for SY-F, < LOQ for SY-G, 0.1% for SY-H, 0.4% for SY-I, < LOQ for SY-J, 0.1% for SY-K and < LOQ for SY-L and SY-M. The proportions of Se^VI^ were: 0.1% for SY-G and < LOQ for the rest of SY products

The proportion of oxyanion Se species (Se^IV^ and Se^VI^) in the 13 analyzed SY samples are illustrated in Fig. 1. The results show that all the tested samples contain only a residual concentration (< 1%) of inorganic Se as Se^IV^ or Se^VI^. These results are in line with previous study [26] and with values reported by EFSA [13, 27] and FDA [17], thus confirming that the Se^IV^ and Se^VI^ used for the enrichment of a yeast growth medium is used by the yeast cells and transformed into other forms. Furthermore, the remaining Se^IV^ and Se^VI^ may be washed out during the production process prior to drying of the SY [28].

## Quantification of Elemental Se (Se^0^) in Seleno-Yeasts (SY)

The results of the quantification of Se^0^ in the 13 SY samples are presented in Table 1. The analysis revealed the presence of Se^0^ in all of the SY samples tested in this study, with an average of 14% (standard deviation; SD:13.2%). The proportion of Se^0^ to total Se ranged between 3.6% and 51.8%. These results corroborate those obtained by Vacchina et al. [20], who found the level of Se^0^ in 7 SY products ranged between 8 and 40%, with an average of 17.4% (SD:10.5%). These proportions can correspond to partially unknown Se species that have been mentioned in different EFSA reports [14–16]. In fact, the proportion of the unknown Se species reported by EFSA varies between 5% [15] and 29% [14], with an average of 17%. According to Jiménez-Lamana et al. [19], the Se mass balance before the quantification of Se^0^ rarely exceeded 90%, thus suggesting the presence of unaccounted forms of Se.

The Se^0^ in SY has been hypothesized to result from the reduction of selenite used in the fermentation process [29]. When yeast cells are exposed to high levels of Se^IV^, they convert it into Se^0^ in an attempt to reduce its toxicity [30, 31]. It is well known that many microorganisms can reduce highly toxic soluble oxyanion Se (Se^IV^ and Se^VI^) to a much less toxic insoluble form, Se^0^ [32]. This transformation was reported to be induced by reductase enzymes [7]. It was observed that the addition of a protein synthesis inhibitor during the culture of yeast cells with Se^IV^ led to the inhibition of the synthesis of Se^0^ [33]. The authors also studied the possibility of generating Se^0^ during the fermentation of yeast and found a significant reduction of the transformation of Se^IV^ into Se^0^ at cold temperatures. They also observed that the process was not influenced by the presence/absence of oxygen and light. Another hypothesis is the transformation of hydrogen selenide, the major intermediate metabolite involved in the synthesis pathway of all forms of Se into Se^0^ via an oxidation reaction [29, 31, 34].

**Fig. 2:**
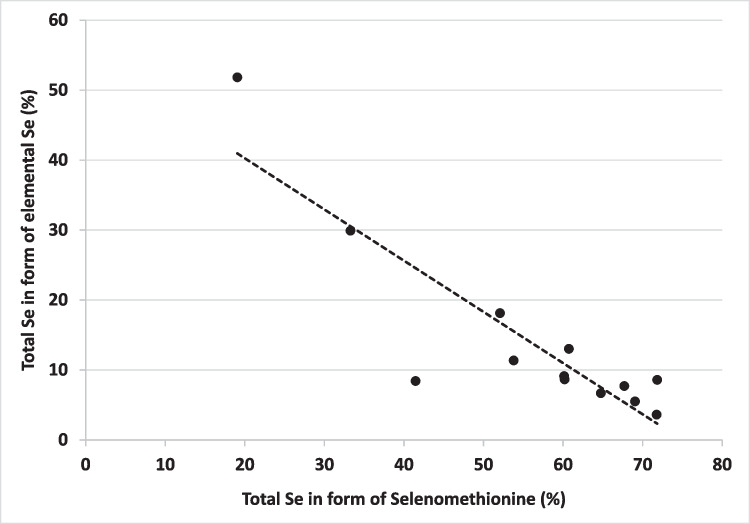
Correlation between the elemental Se content and selenomethionine content in Seleno-yeast (SY). Y = -0.732 + 0.549 (R^2^ = 0.781)

We also observed a negative correlation between the proportion of Se^0^ and SeMet in SY, with a coefficient of 0.78 (*P* < 0.001; Fig. 2). High levels of Se^IV^ can reduce the growth of yeast cells and induce damage, as shown in the study of Rajashree and Muthukumar, [35]. The toxicity of the Se^IV^ in SY was reported to be the result of such different physiological effects as: a genotoxicity effect, mitochondrial alteration and redox imbalance [34]. Thus, high levels of Se^IV^ in the yeast growth media decrease their ability to synthesize SeMet and lead to a reduction of the transformation of Se^IV^ into Se^0^. This could explain the negative correlation between SeMet and Se^0^. Hence, the proportion of Se^0^ in SY could be used as an additional indicator of the quality of SY products.

With respect to the bioavailability of Se^0^, it was shown that Se^IV^ and Se^0^ have a similar bioavailability in different tissues (muscle, liver and plasma) of rats and can cause an equal regulation of plasma selenoprotein P, and it was thus concluded that both forms of Se were equally absorbed, distributed, metabolized and excreted [36]. Moreover, Galbraith et al. [21] reported that rumen microorganisms contributed to the conversion of dietary Se^IV^ into Se^0^, a non-bioavailable Se form, thus leading to its eventual loss in feces.

## Quantification of SeMet in Seleno-Yeasts

The proportions of SeMet in the 13 tested SY are summarized in Fig. 1. These results indicate a SeMet content ranging from 19 to 71.8%, with an average of 55.8% (SD:15.9%). The SeMet incorporation in SY is non-specific and involves the replacement of about 30% of methionine with selenomethionine [6, 37]. In a previous study, 12 SY samples of eight commercial products were collected and analyzed to establish the SeMet content [38], and the authors showed a high variation of SeMet, with an average of 60% total Se. The analysis of 11 samples from different batches of five different producers sourced in the EU and the USA revealed an SeMet content ranging from 24.8% to 69.7% of the total Se [39]. Bierla et al. [22] studied the composition of three SY products and found that the SeMet content varied between 60 and 81%. Rayman reviewed the composition of 7 commercial SY and found a content of 55–75% of Se as SeMet [4]. The variability of SeMet has been reported extensively and attributed to the yeast strain and the fermentation process [5, 9, 28]. Different factors can in fact affect the transformation of Se into SeMet in growing yeast, including the quantity of the sodium selenite used in the medium, the pH, yeast strain, etc. Most of the methodological developments concerning Se analysis have been focused on the quantitative determination of SeMet, because it is the driver of SY bio-efficacy [23]. A SeMet content above a certain value has been accepted as a crucial parameter of the quality control of SY products [6]. EFSA has reported that the SY authorized for animal feeds should contain at least 63%, and in some cases 70%, of Se as SeMet. In the current study, the SeMet proportion was below 63% in 8 of the analyzed SY samples and below 70% in 11 SY samples. Moreover, this proportion represented only one third, or even one-fifth, of the total Se in certain SY. The low proportion of SeMet could be explained by the transformation of oxyanion Se into Se^0^, rather than into SeMet, during the fermentation process, as mentioned above. In addition, the degradation of SeMet could also explain the low content of SeMet in SY [9]. In fact, the authors detected more SeMet after the addition of the reducing reagent, thus indicating the presence of oxidized forms of SeMet, as previously reported Gammelgaard et al. by [40]. Overall, the results of the SeMet determination in SY products confirm the difficulties of guaranteeing an exact and reliable content of SeMet in commercial SY products, which can explain the variable efficacy of SY when used as feed additives [3, 38].

## Quantification of SeCys in Seleno-Yeasts

The results pertaining to the proportion of SeCys in the SY samples are reported in Fig. 1. The total Se in the form of SeCys, varied between 1.2 and 6.6%, with an average of 3.8% (SD: 1.7%). In 2013, 15–30% of total Se as SeCys was estimated in SY, which could represent 1/3 of the total Se [6]. Moreover, EFSA reported that the SeCys in SY was 13–26% of the total Se [15, 16, 27]. However, the findings of the current experiment reveal that the SeCys content in SY is low and thus corroborates the result obtained by Bierla et al. [22], who found an average of 4% of SeCys in 3 SY samples from different producers. The results of the present study are comparable with those of Bierla et al. [22] and those reported by EFSA [41] with 2 – 4% of the total Se being identified as SeCys. The SeCys in SY is found in the water-soluble fraction [42]. Seleno-yeast can incorporate SeCys in the proteins of the yeast proteome, despite the absence of the SeCys insertion sequence (SECIS) in the genome [14]. The SeCys proportion in SY is lower than the SeMet one because cysteine is less abundant than methionine [6, 43, 44], and for those sulfur homologues the degree of substitution of cysteine is lower than that of methionine [24]. Finally, the bio-efficacy of the SeCys form for animals is low and similar to selenite because, unlike SeMet, this Se form does not represent a storage form of Se and cannot be used as it is for selenoprotein synthesis, due to the complex and specific translation machinery, which uses a hydrogen selenide intermediate [3, 45, 46].

## The proportion of Inorganic and Organic Se in SY

The proportion of inorganic Se (iSe) represents the sum of the analyzed Se^0^, Se^IV^ and Se^VI^, while the proportion of organic (oSe) was computed as the difference between the total Se and iSe. The results showed that the proportion of iSe ranged between 3.8 and 51.8%, with an average of 14.2%, and the proportion of oSe ranged between 48 and 96%, with an average of 85.8% (Table 2). FDA and EFSA currently regulate the SY that has to be used in animal feeds so the iSe content is less than 2% of the total Se (FDA 2022). Organic Se is a generic term that covers different chemical Se species in which the Se atom is chemically bound an integral part of an organic molecule [24]. In fact, to date, only Se^IV^ and Se^VI^ have been considered as iSe species in SY, and the remaining Se metabolites have been presumed to be oSe [47]. This is the result of the assumption that the metabolism of the sodium selenite utilized in the process of fermentation leads to the formation of a bond between Se and carbon, thereby producing several organoselenium compounds [24]. Moreover, different studies performed to characterize the Se species of SY have only found organic Se compounds [10, 48]. However, it should be pointed out that those studies only analyzed the water-soluble fraction of SY, and Se^0^ was not detected because it is a water-insoluble species. Elemental Se is also an iSe that has already been detected [19, 33, 49, 50], but its accurate quantification in SY has not been possible until recently [20]. The presence of a significant quantity of Se^0^, and consequently of iSe, helps to explain the bio-efficacy results of SY when used as a feed additive [3]. The quantification of an important amount of iSe in SY has highlighted the need to review the proportion of organic and inorganic Se currently specified by SY suppliers. The increased bio-efficacy of oSe, in comparison to iSe, has been reported for different animal species, including broilers, laying hens, growing pigs, sows as well as dairy and beef cattle [2, 51–55].

**Table 2:** The organic Se and inorganic Se contents of 13 fresh commercial Seleno-yeast (SY) samples

SY samples	Organic Se content as % of total Se	Inorganic Se content as % of total Se
SY-A	91.3	8.7
SY-B	94.2	5.8
SY-C	92.1	7.9
SY-D	96.2	3.8
SY-E	86.9	13.1
SY-F	48.2	51.8
SY-G	70.0	30.0
SY-H	91.3	8.7
SY-I	90.5	9.5
SY-J	88.6	11.4
SY-K	91.5	8.5
SY-L	81.9	18.1
SY-M	93.3	6.7
Average	85.8	14.2
Standard deviation, SD	13.2	13.2

Inorganic Se (%) = ((Sum of the elemental Se + Selenite + Selenate)/ Total Se) * 100

Organic Se content (%) = Total Se (100%) – Inorganic Se (%)

## Other Se Compounds

Other Se compounds are defined from the difference between the total analyzed Se and the sum of the analyzed Se species, as presented in Fig. 1. The proportion of these undefined Se species is between 12.9 and 48%, with an average of 26.3% (SD: 8.4%). SY has been reported to contain more than 100 unique Se species [56] or even 180 [10]. To date, only SeMet has been clearly described and has been proven to be the driver of the bio-efficacy of SY [3]. The possible roles and effects of these other Se compounds in animals/poultry still require further investigation, although, on the basis of the current knowledge, it can be concluded that, in most cases, their bio-efficacy is not so different from that of sodium selenite [2, 3]. Simon et al. [57], in a test on broiler chickens, compared sodium selenite and two SY with 56.7% and 63% of SeMet, respectively, and concluded that the two SY were not the same and their bioavailability depended on their SeMet content. Van Beirendonck et al. [58] also showed that the Se content in the muscles of broilers was significantly higher in broilers fed SY with 69% of SeMet than broilers fed SY with 26% of SeMet.

Indeed, Se^IV^, Se^VI^, and even SeCys are ineffective in increasing the Se concentration in muscle tissue and building Se reserves in monogastric animals, as shown by Deagen et al. [45] for rats and by De Marco et al. [3] for broilers, and only SeMet can non-specifically be incorporated into proteins in place of methionine [4, 8]. The higher bio-efficacy of SeMet has mainly been observed in farm animals’ studies, such us in the muscles of chickens [3, 52, 58], the eggs of laying hens [54, 59, 60], the muscles of growing pigs [53, 61, 62], in sow milk [63] and in milk and beef cattle [55, 64, 65].

The development of these new analytical methods is another step forward in achieving the mass balance of the Se speciation in SY. However, it is still not possible to conclude that the mass balance is complete. It is believed that the remaining Se species are water-soluble metabolites, with a high probability of being oSe, but the presence of other iSe species, in addition to Se^0^, Se^IV^ and Se^VI^, cannot be excluded. The aforementioned analytical method may also lead to an underestimation of SeMet and/or the SeCys content, due to a low extraction or their oxidation after extraction during the analysis. The bio-efficacy of such an underestimated fraction should be considered, due to its low bio-accessibility for animals [66]. In addition, significant variation among different SY products was confirmed in this study. In fact, SY production is a complex process based on the growth and multiplication of the yeast and enrichment of the Se content by use of inorganic Sodium Selenite or Selenate. However, as inorganic Se is toxic for living organisms, the incorporation of Se into the yeasts is a delicate process to avoid killing the yeasts. Overall, the speciation of SY samples has revealed the presence of large amounts of Se^0^, which, consequently, increase the observed proportion of iSe in SY. The analysis has also shown a high variability of SeMet in the SY sources used in animal nutrition.

## Conclusion

The recent developments in advanced analytical methodologies allow accurate, reliable and more comprehensive determinations of the Se composition of SY to be made. These analyses have revealed the presence of an inorganic Se species: Se^0^, which explains a great deal about the previously unknown Se species. These findings have revealed that the SY products used in animal nutrition contain, on average far less than 97% of total Se as organic Se, and consequently, the proportion of inorganic Se in SY products needs to be revised. According to these findings, the characterization of SY as a full organic form of Se is now questionable, and end users and the industry now have the opportunity to make more meaningful choices.

## Data Availability

The datasets generated and/or analyzed during the current study are available from the corresponding author on reasonable request.
